# Supplementary material for: Modeling glioblastoma heterogeneity as a dynamic network of cell states
Source: Mol Syst Biol. 2021 Sep 16;17(9):e10105. doi: 10.15252/msb.202010105 (PMC8444284; doi:10.15252/msb.202010105)
Supplement: Supplementary file 6 — Source Data for Figure 5 [file MSB-17-e10105-s004.zip › Figure5A_sourcedata/GSEA_3071/nosigdb_stateIV.GseaPreranked.1622795164626/gsea_report_for_na_pos_1622795164626.html]

Report for na\_pos 1622795164626 [GSEA]

| GS  follow link to MSigDB | GS DETAILS | SIZE | ES | NES | NOM p-val | FDR q-val | FWER p-val | RANK AT MAX | LEADING EDGE || 1 | SUVA\_NPC1\_PMID\_31327527 | Details ... | 37 | 0.77 | 3.44 | 0.000 | 0.000 | 0.000 | 719 | tags=70%, list=14%, signal=81% |
| 2 | RICHARDS\_NEFTEL\_CELL\_2019\_NPC1 | Details ... | 37 | 0.77 | 3.37 | 0.000 | 0.000 | 0.000 | 719 | tags=70%, list=14%, signal=81% |
| 3 | LARSSON\_STATE\_6 | Details ... | 73 | 0.64 | 3.31 | 0.000 | 0.000 | 0.000 | 913 | tags=62%, list=17%, signal=73% |
| 4 | RICHARDS\_NOWAKOWSKI\_SCIENCE\_2017\_OPC\_UPREG | Details ... | 237 | 0.51 | 3.26 | 0.000 | 0.000 | 0.000 | 796 | tags=45%, list=15%, signal=50% |
| 5 | RICHARDS\_NOWAKOWSKI\_SCIENCE\_2017\_NIN1\_UPREG | Details ... | 117 | 0.58 | 3.26 | 0.000 | 0.000 | 0.000 | 611 | tags=42%, list=12%, signal=46% |
| 6 | RICHARDS\_ZHONG\_NATURE\_2018\_OPC\_UPREG | Details ... | 59 | 0.66 | 3.21 | 0.000 | 0.000 | 0.000 | 559 | tags=56%, list=11%, signal=62% |
| 7 | RICHARDS\_VERHAAK\_CANCERCELL\_2010\_PRONEURAL | Details ... | 90 | 0.58 | 3.10 | 0.000 | 0.000 | 0.000 | 438 | tags=39%, list=8%, signal=42% |
| 8 | RICHARDS\_NOWAKOWSKI\_SCIENCE\_2017\_IPC\_NEN1\_UPREG | Details ... | 179 | 0.51 | 3.05 | 0.000 | 0.000 | 0.000 | 1422 | tags=64%, list=27%, signal=84% |
| 9 | RICHARDS\_NOWAKOWSKI\_SCIENCE\_2017\_IN\_CTX\_CGE2\_UPREG | Details ... | 135 | 0.52 | 3.03 | 0.000 | 0.000 | 0.000 | 1140 | tags=54%, list=22%, signal=67% |
| 10 | RICHARDS\_NOWAKOWSKI\_SCIENCE\_2017\_ASTROCYTE\_UPREG | Details ... | 355 | 0.45 | 3.01 | 0.000 | 0.000 | 0.000 | 1110 | tags=50%, list=21%, signal=59% |
| 11 | RICHARDS\_NOWAKOWSKI\_SCIENCE\_2017\_NIN3\_UPREG | Details ... | 104 | 0.54 | 2.97 | 0.000 | 0.000 | 0.000 | 567 | tags=38%, list=11%, signal=42% |
| 12 | GAROFANO\_IAVARONE\_NEU\_SUBTYPE | Details ... | 145 | 0.50 | 2.91 | 0.000 | 0.000 | 0.000 | 1310 | tags=57%, list=25%, signal=74% |
| 13 | NB\_KI\_CLUSTER4 | Details ... | 70 | 0.58 | 2.89 | 0.000 | 0.000 | 0.000 | 725 | tags=51%, list=14%, signal=59% |
| 14 | SINGH\_SOX2\_UPREGULATED\_TRANSCRIPTS\_PMID\_28122245 | Details ... | 255 | 0.45 | 2.87 | 0.000 | 0.000 | 0.000 | 734 | tags=36%, list=14%, signal=40% |
| 15 | NB\_KI\_CLUSTER2 | Details ... | 164 | 0.48 | 2.86 | 0.000 | 0.000 | 0.000 | 1515 | tags=66%, list=29%, signal=89% |
| 16 | RICHARDS\_NOWAKOWSKI\_SCIENCE\_2017\_IN\_CTX\_CGE1\_UPREG | Details ... | 163 | 0.47 | 2.83 | 0.000 | 0.000 | 0.000 | 802 | tags=38%, list=15%, signal=43% |
| 17 | SUVA\_NPC2\_PMID\_31327527 | Details ... | 30 | 0.68 | 2.83 | 0.000 | 0.000 | 0.000 | 701 | tags=63%, list=13%, signal=73% |
| 18 | RICHARDS\_NEFTEL\_CELL\_2019\_NPC2 | Details ... | 30 | 0.68 | 2.81 | 0.000 | 0.000 | 0.000 | 701 | tags=63%, list=13%, signal=73% |
| 19 | RICHARDS\_NOWAKOWSKI\_SCIENCE\_2017\_NEN\_EARLY2\_UPREG | Details ... | 277 | 0.44 | 2.81 | 0.000 | 0.000 | 0.000 | 1453 | tags=58%, list=27%, signal=76% |
| 20 | VANGRONINGEN\_ADRN | Details ... | 186 | 0.45 | 2.79 | 0.000 | 0.000 | 0.000 | 785 | tags=42%, list=15%, signal=48% |
| 21 | RICHARDS\_NOWAKOWSKI\_SCIENCE\_2017\_IN\_CTX\_MGE1\_UPREG |  | 198 | 0.44 | 2.71 | 0.000 | 0.000 | 0.000 | 701 | tags=33%, list=13%, signal=36% |
| 22 | RICHARDS\_NOWAKOWSKI\_SCIENCE\_2017\_NIN5\_UPREG |  | 160 | 0.46 | 2.68 | 0.000 | 0.000 | 0.000 | 615 | tags=33%, list=12%, signal=36% |
| 23 | DOROSZKO\_U3054MG\_CLUSTER\_0 |  | 55 | 0.55 | 2.67 | 0.000 | 0.000 | 0.000 | 844 | tags=60%, list=16%, signal=71% |
| 24 | RICHARDS\_NOWAKOWSKI\_SCIENCE\_2017\_EN\_PFC1\_UPREG |  | 491 | 0.38 | 2.62 | 0.000 | 0.000 | 0.000 | 1270 | tags=43%, list=24%, signal=51% |
| 25 | RICHARDS\_NOWAKOWSKI\_SCIENCE\_2017\_NIN4\_UPREG |  | 94 | 0.47 | 2.61 | 0.000 | 0.000 | 0.000 | 623 | tags=33%, list=12%, signal=37% |
| 26 | POLLEN\_OUTERRADIALGLIA\_PMID\_26406371 |  | 43 | 0.57 | 2.59 | 0.000 | 0.000 | 0.000 | 913 | tags=58%, list=17%, signal=70% |
| 27 | LARSSON\_STATE\_2 |  | 55 | 0.55 | 2.59 | 0.000 | 0.000 | 0.000 | 1199 | tags=65%, list=23%, signal=84% |
| 28 | DOROSZKO\_U3180MG\_CLUSTER\_2 |  | 270 | 0.40 | 2.59 | 0.000 | 0.000 | 0.000 | 1546 | tags=57%, list=29%, signal=76% |
| 29 | SUVA\_AC\_PMID\_31327527 |  | 30 | 0.62 | 2.59 | 0.000 | 0.000 | 0.000 | 521 | tags=50%, list=10%, signal=55% |
| 30 | RICHARDS\_NOWAKOWSKI\_SCIENCE\_2017\_IN\_CTX\_MGE2\_UPREG |  | 153 | 0.44 | 2.59 | 0.000 | 0.000 | 0.000 | 1114 | tags=45%, list=21%, signal=55% |
| 31 | OUTERRADIALGLIA |  | 43 | 0.57 | 2.59 | 0.000 | 0.000 | 0.000 | 913 | tags=58%, list=17%, signal=70% |
| 32 | RICHARDS\_NOWAKOWSKI\_SCIENCE\_2017\_MGE\_IPC3\_UPREG |  | 87 | 0.48 | 2.57 | 0.000 | 0.000 | 0.000 | 532 | tags=36%, list=10%, signal=39% |
| 33 | TISIG\_PMID\_32649888 |  | 37 | 0.58 | 2.57 | 0.000 | 0.000 | 0.000 | 1384 | tags=81%, list=26%, signal=109% |
| 34 | RICHARDS\_NOWAKOWSKI\_SCIENCE\_2017\_VRG\_UPREG |  | 274 | 0.39 | 2.55 | 0.000 | 0.000 | 0.000 | 1001 | tags=42%, list=19%, signal=49% |
| 35 | RICHARDS\_NEFTEL\_CELL\_2019\_AC |  | 30 | 0.62 | 2.55 | 0.000 | 0.000 | 0.000 | 521 | tags=50%, list=10%, signal=55% |
| 36 | OLSEN\_ADRENERGIC |  | 55 | 0.51 | 2.48 | 0.000 | 0.000 | 0.000 | 725 | tags=45%, list=14%, signal=52% |
| 37 | RICHARDS\_NOWAKOWSKI\_SCIENCE\_2017\_EN\_PFC3\_UPREG |  | 171 | 0.41 | 2.45 | 0.000 | 0.000 | 0.000 | 844 | tags=37%, list=16%, signal=43% |
| 38 | LARSSON\_STATE\_3 |  | 48 | 0.51 | 2.42 | 0.000 | 0.000 | 0.000 | 506 | tags=40%, list=10%, signal=43% |
| 39 | RICHARDS\_NOWAKOWSKI\_SCIENCE\_2017\_IN\_STR\_UPREG |  | 102 | 0.43 | 2.41 | 0.000 | 0.000 | 0.000 | 569 | tags=29%, list=11%, signal=32% |
| 40 | RICHARDS\_NOWAKOWSKI\_SCIENCE\_2017\_IPC\_NEN3\_UPREG |  | 155 | 0.40 | 2.41 | 0.000 | 0.000 | 0.000 | 590 | tags=30%, list=11%, signal=33% |
| 41 | RICHARDS\_ZHONG\_NATURE\_2018\_ASTROCYTES\_UPREG |  | 188 | 0.39 | 2.40 | 0.000 | 0.000 | 0.000 | 559 | tags=31%, list=11%, signal=33% |
| 42 | RICHARDS\_NOWAKOWSKI\_SCIENCE\_2017\_EN\_V1\_1\_UPREG |  | 485 | 0.34 | 2.39 | 0.000 | 0.000 | 0.000 | 1428 | tags=44%, list=27%, signal=55% |
| 43 | ALLEN\_113\_ASTROL1FGFR3FOS |  | 19 | 0.65 | 2.37 | 0.000 | 0.000 | 0.000 | 518 | tags=58%, list=10%, signal=64% |
| 44 | RICHARDS\_NOWAKOWSKI\_SCIENCE\_2017\_IPC\_NEN1\_UPREG\_1 |  | 87 | 0.45 | 2.36 | 0.000 | 0.000 | 0.000 | 1292 | tags=55%, list=24%, signal=72% |
| 45 | ALLEN\_112\_ASTROL1FGFR3MT1G |  | 22 | 0.64 | 2.35 | 0.000 | 0.000 | 0.000 | 470 | tags=50%, list=9%, signal=55% |
| 46 | SUVA\_OPC\_PMID\_31327527 |  | 27 | 0.59 | 2.35 | 0.000 | 0.000 | 0.000 | 555 | tags=48%, list=10%, signal=54% |
| 47 | RICHARDS\_NEFTEL\_CELL\_2019\_OPC |  | 27 | 0.59 | 2.35 | 0.000 | 0.000 | 0.000 | 555 | tags=48%, list=10%, signal=54% |
| 48 | ALLEN\_114\_OPCL1\_6MYT1 |  | 15 | 0.67 | 2.24 | 0.000 | 0.000 | 0.004 | 470 | tags=60%, list=9%, signal=66% |
| 49 | IVY\_IT |  | 45 | 0.49 | 2.22 | 0.000 | 0.000 | 0.004 | 1304 | tags=62%, list=25%, signal=82% |
| 50 | IVY\_CT |  | 141 | 0.37 | 2.22 | 0.000 | 0.000 | 0.004 | 575 | tags=33%, list=11%, signal=36% |
| 51 | DEPRETER\_LOW\_NB\_RISK |  | 16 | 0.65 | 2.22 | 0.000 | 0.000 | 0.004 | 294 | tags=38%, list=6%, signal=40% |
| 52 | RICHARDS\_NOWAKOWSKI\_SCIENCE\_2017\_NEN\_EARLY1\_UPREG |  | 387 | 0.33 | 2.19 | 0.000 | 0.000 | 0.005 | 1430 | tags=47%, list=27%, signal=59% |
| 53 | TIISIG\_PMID\_32649888 |  | 26 | 0.55 | 2.18 | 0.002 | 0.000 | 0.007 | 243 | tags=31%, list=5%, signal=32% |
| 54 | GAROFANO\_IAVARONE\_MTC\_SUBTYPE |  | 104 | 0.39 | 2.14 | 0.000 | 0.000 | 0.008 | 1340 | tags=50%, list=25%, signal=66% |
| 55 | RICHARDS\_NOWAKOWSKI\_SCIENCE\_2017\_NIN2\_UPREG |  | 278 | 0.33 | 2.12 | 0.000 | 0.000 | 0.011 | 706 | tags=24%, list=13%, signal=26% |
| 56 | RICHARDS\_NOWAKOWSKI\_SCIENCE\_2017\_EN\_V1\_2\_UPREG |  | 437 | 0.31 | 2.12 | 0.000 | 0.000 | 0.011 | 1417 | tags=43%, list=27%, signal=54% |
| 57 | IVY\_LE |  | 35 | 0.48 | 2.07 | 0.000 | 0.000 | 0.018 | 1206 | tags=54%, list=23%, signal=70% |
| 58 | RICHARDS\_NOWAKOWSKI\_SCIENCE\_2017\_NEN\_LATE\_UPREG |  | 190 | 0.34 | 2.07 | 0.000 | 0.000 | 0.018 | 1152 | tags=37%, list=22%, signal=46% |
| 59 | DOROSZKO\_U3054MG\_CLUSTER\_5 |  | 76 | 0.39 | 2.07 | 0.000 | 0.000 | 0.018 | 1257 | tags=53%, list=24%, signal=68% |
| 60 | DOROSZKO\_U3180MG\_CLUSTER\_1 |  | 18 | 0.56 | 2.06 | 0.002 | 0.000 | 0.019 | 1014 | tags=61%, list=19%, signal=75% |
| 61 | DOROSZKO\_U3013MG\_CLUSTER\_0 |  | 26 | 0.51 | 2.06 | 0.002 | 0.000 | 0.019 | 234 | tags=42%, list=4%, signal=44% |
| 62 | RICHARDS\_VERHAAK\_CANCERCELL\_2010\_CLASSICAL |  | 80 | 0.37 | 2.01 | 0.000 | 0.001 | 0.036 | 923 | tags=36%, list=17%, signal=43% |
| 63 | DOROSZKO\_U3054MG\_CLUSTER\_8 |  | 327 | 0.30 | 2.01 | 0.000 | 0.001 | 0.036 | 1663 | tags=49%, list=31%, signal=67% |
| 64 | KRONA\_STEM |  | 15 | 0.61 | 1.97 | 0.002 | 0.001 | 0.050 | 214 | tags=40%, list=4%, signal=42% |
| 65 | ALMSTEDT\_ALK |  | 43 | 0.43 | 1.97 | 0.000 | 0.001 | 0.051 | 611 | tags=40%, list=12%, signal=44% |
| 66 | DOROSZKO\_U3054MG\_CLUSTER\_7 |  | 21 | 0.53 | 1.95 | 0.002 | 0.001 | 0.056 | 645 | tags=43%, list=12%, signal=49% |
| 67 | GANDAL\_ALCOHOLADDICTION\_DN\_PMID29439242 |  | 49 | 0.43 | 1.95 | 0.002 | 0.001 | 0.056 | 946 | tags=41%, list=18%, signal=49% |
| 68 | ALLEN\_111\_ASTROL1\_6FGFR3ETNPPL |  | 23 | 0.51 | 1.94 | 0.000 | 0.001 | 0.065 | 509 | tags=39%, list=10%, signal=43% |
| 69 | DOROSZKO\_PERIVASCULAR\_INVASION |  | 32 | 0.46 | 1.92 | 0.000 | 0.002 | 0.079 | 180 | tags=34%, list=3%, signal=35% |
| 70 | RICHARDS\_NOWAKOWSKI\_SCIENCE\_2017\_EN\_V1\_3\_UPREG |  | 463 | 0.27 | 1.85 | 0.000 | 0.003 | 0.145 | 1307 | tags=37%, list=25%, signal=44% |
| 71 | DOROSZKO\_U3180MG\_CLUSTER\_5 |  | 23 | 0.49 | 1.84 | 0.002 | 0.003 | 0.152 | 456 | tags=35%, list=9%, signal=38% |
| 72 | ALMSTEDT\_RISK |  | 28 | 0.45 | 1.79 | 0.007 | 0.005 | 0.237 | 294 | tags=25%, list=6%, signal=26% |
| 73 | RICHARDS\_NOWAKOWSKI\_SCIENCE\_2017\_MGE\_RG1\_UPREG |  | 211 | 0.28 | 1.79 | 0.000 | 0.005 | 0.237 | 602 | tags=24%, list=11%, signal=26% |
| 74 | WENG\_STEMNESS\_PMID\_30982771 |  | 24 | 0.46 | 1.78 | 0.005 | 0.005 | 0.250 | 739 | tags=46%, list=14%, signal=53% |
| 75 | OLSEN\_SCPLIKE |  | 61 | 0.35 | 1.76 | 0.007 | 0.006 | 0.294 | 645 | tags=38%, list=12%, signal=42% |
| 76 | GAROFANO\_IAVARONE\_LIST\_OF\_NEURAL\_PROGENITOR\_MARKER\_GENES |  | 75 | 0.33 | 1.72 | 0.000 | 0.008 | 0.381 | 339 | tags=23%, list=6%, signal=24% |
| 77 | DOROSZKO\_U3054MG\_CLUSTER\_10 |  | 148 | 0.27 | 1.64 | 0.000 | 0.015 | 0.596 | 1113 | tags=33%, list=21%, signal=41% |
| 78 | GANDAL\_BIPOLAR\_DN\_PMID29439242 |  | 18 | 0.47 | 1.62 | 0.036 | 0.017 | 0.650 | 530 | tags=44%, list=10%, signal=49% |
| 79 | WANG\_PRONEURAL\_PMID\_28697342 |  | 19 | 0.43 | 1.59 | 0.047 | 0.021 | 0.721 | 565 | tags=26%, list=11%, signal=29% |
| 80 | TF\_CENSUS\_TAIPALE |  | 479 | 0.22 | 1.54 | 0.000 | 0.028 | 0.816 | 1290 | tags=33%, list=24%, signal=39% |
| 81 | RICHARDS\_CAHOY\_JNEUROSCI\_2011\_ASTRO\_MATURE |  | 316 | 0.23 | 1.52 | 0.000 | 0.032 | 0.855 | 944 | tags=26%, list=18%, signal=29% |
| 82 | WANG\_CLASSICAL\_PMID\_28697342 |  | 21 | 0.41 | 1.51 | 0.060 | 0.033 | 0.869 | 1384 | tags=57%, list=26%, signal=77% |
| 83 | NB\_KI\_CLUSTER8 |  | 152 | 0.25 | 1.51 | 0.009 | 0.033 | 0.871 | 1992 | tags=61%, list=38%, signal=94% |
| 84 | DOROSZKO\_U3013MG\_CLUSTER\_3 |  | 80 | 0.29 | 1.50 | 0.025 | 0.034 | 0.889 | 909 | tags=31%, list=17%, signal=37% |
| 85 | PARADA\_T1\_DN\_26461091 |  | 31 | 0.36 | 1.49 | 0.054 | 0.037 | 0.905 | 1418 | tags=58%, list=27%, signal=79% |
| 86 | NB\_KI\_CLUSTER6 |  | 201 | 0.23 | 1.40 | 0.014 | 0.065 | 0.990 | 2009 | tags=54%, list=38%, signal=84% |
| 87 | RICHARDS\_CAHOY\_JNEUROSCI\_2014\_MOG\_POS |  | 383 | 0.20 | 1.34 | 0.003 | 0.086 | 0.999 | 1070 | tags=29%, list=20%, signal=33% |
| 88 | DOROSZKO\_U3054MG\_CLUSTER\_3 |  | 138 | 0.23 | 1.32 | 0.064 | 0.097 | 1.000 | 1259 | tags=37%, list=24%, signal=47% |
| 89 | PARADA\_T2\_DN\_26461091 |  | 32 | 0.32 | 1.31 | 0.131 | 0.100 | 1.000 | 1466 | tags=53%, list=28%, signal=73% |
| 90 | GANDAL\_SCHIZOPHRENIA\_UP\_PMID29439242 |  | 65 | 0.25 | 1.29 | 0.099 | 0.114 | 1.000 | 452 | tags=20%, list=9%, signal=22% |
| 91 | ALLEN\_115\_OLIGOL4\_6MOBPCOL18A1 |  | 16 | 0.37 | 1.21 | 0.240 | 0.178 | 1.000 | 257 | tags=25%, list=5%, signal=26% |
| 92 | LARSSON\_STATE\_5 |  | 56 | 0.24 | 1.17 | 0.220 | 0.221 | 1.000 | 1049 | tags=32%, list=20%, signal=40% |
| 93 | RICHARDS\_LIDDELOW\_NATURE\_2017\_A1\_REACTIVE\_ASTROCYTES |  | 22 | 0.30 | 1.14 | 0.295 | 0.250 | 1.000 | 188 | tags=14%, list=4%, signal=14% |
| 94 | GANDAL\_SCHIZOPHRENIA\_DN\_PMID29439242 |  | 19 | 0.30 | 1.10 | 0.347 | 0.306 | 1.000 | 188 | tags=21%, list=4%, signal=22% |
| 95 | GANDAL\_DEPRESSION\_DN\_PMID29439242 |  | 33 | 0.19 | 0.84 | 0.701 | 0.784 | 1.000 | 1201 | tags=36%, list=23%, signal=47% |
| 96 | NB\_KI\_CLUSTER22 |  | 77 | 0.15 | 0.77 | 0.845 | 0.866 | 1.000 | 760 | tags=18%, list=14%, signal=21% |
| 97 | NB\_KI\_CLUSTER7 |  | 98 | 0.10 | 0.52 | 0.998 | 0.989 | 1.000 | 4676 | tags=99%, list=88%, signal=836% |
Table: Gene sets enriched in phenotype **na **[plain text format]****
